# Cross-platform, multi-cohort identification of a caries microbial signature

**DOI:** 10.1080/20002297.2026.2737699

**Published:** 2026-09-28

**Authors:** Anders Esberg, Daniel Jönsson, Peter Persson, Linda Eriksson, Simon Haworth, Ingegerd Johansson

**Affiliations:** a Department of Odontology, Umeå University, Sweden; b Department of Clinical Sciences, Lund University, Malmö, Sweden; c Public Dental Service of Skåne, Lund, Sweden; d Bristol Dental School, University of Bristol, Bristol, UK

**Keywords:** Oxford nanopore sequencing, metagenomic, oral microbiome, dental health, caries, risk score

## Abstract

**Objective:**

To identify a robust salivary microbial signature associated with caries using a cross-platform, multi-cohort design.

**Design:**

Full-length 16S rRNA gene sequencing (ONT) and shotgun metagenomics (MET) were performed in a derivation cohort with matched data (*n* = 463). Associations between the oral microbiome and caries were evaluated using confounder-adjusted linear models with nested cross-validation. Identified species were incorporated into microbial risk scores and tested in two independent ONT-based cohorts (*n* = 3,457 and *n* = 215).

**Results:**

ONT and MET shared community structure despite differences in sequencing depth and detection sensitivity. Inter-individual ecological distances were moderately correlated (Spearman *ρ* = 0.48), with significant Procrustes alignment (r = 0.60), and mean relative abundances of shared species were strongly correlated (*ρ* = 0.75), but agreement at the individual species level was more variable. Ten species were consistently associated with caries across platforms and analytical approaches. Risk scores based on these species were strongly associated with caries burden in the derivation cohort (R^2^ = 0.46 for MET; R^2^ = 0.42 for ONT) and replicated in independent cohorts (mean R^2^ = 0.27 and 0.10). Detection- and abundance-based scores performed similarly and combining them provided minimal additional predictive value. Detection-based scores showed a slightly greater incremental contribution to model fit. Leave-one-out analyses confirmed that associations were not strongly driven by individual species.

**Conclusions:**

Key ecological features of the oral microbiome are reproducible across sequencing platforms. The microbial signature of caries can be summarised using a detection-based risk score which may have potential uses in caries risk assessment.

## Key messages

- Despite substantial differences in sequencing depth and taxonomic sensitivity, Oxford Nanopore full-length 16S rRNA sequencing (ONT) and shotgun metagenomics (MET) showed agreement in overall community structure and dominant taxa, although concordance at the individual species level was more variable.
- Reproducible associations of multiple microbial species with caries across sequencing platforms, analytical approaches, and independent cohorts support a polymicrobial ecological signature rather than single-pathogen detection.
- A detection-based microbial risk score performed as well as or better than abundance-based models and replicated across independent cohorts, suggesting that presence/absence of key taxa may provide a practical and platform-independent approach for caries risk assessment.

## Graphical abstract

**Figure:**
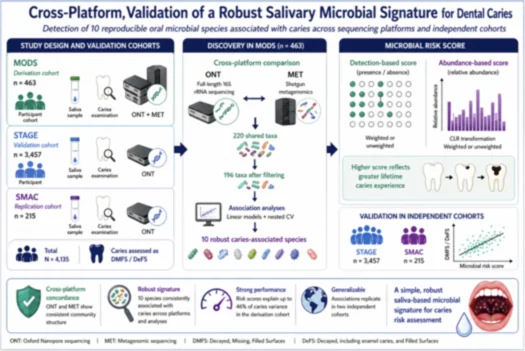

## Introduction

The oral cavity harbours a complex and dynamic microbial ecosystem central to both oral and systemic health [1,2]. A balanced oral microbiome supports functions including nutrient metabolism, immune modulation, and protection against opportunistic pathogens [3]. Disruption of this equilibrium, through factors such as diet, oral hygiene, medication, and systemic disease, can lead to microbial dysbiosis [4], which is associated with prevalent oral diseases, including dental caries, and broader systemic conditions [5,6].

High-throughput sequencing technologies have transformed our ability to characterise microbial communities and their relationship to disease. Shotgun metagenomic sequencing (MET) enables comprehensive taxonomic and functional profiling but is associated with higher cost, computational demands, and host DNA contamination. In contrast, full-length 16S rRNA gene sequencing using Oxford Nanopore Technologies (ONT) provides species-level resolution with lower cost and streamlined workflows, making it well suited for large-scale epidemiological studies.

Amplicon-based 16S sequencing and whole-genome metagenomics have each advanced our understanding of oral microbial ecology. However, most epidemiological studies rely on single cohorts and platforms. Differences in sampling, sequencing, bioinformatics, and phenotype definition can influence reported associations [7], making it unclear whether findings reflect generalisable biology or platform-specific effects. Identifying microbial signatures reproducible across platforms, definitions, and populations is therefore critical for clinical and epidemiological translation.

In this study, we used ONT-based microbial profiling as the primary framework to investigate associations between the oral microbiome and dental caries. The primary aim of the present study was to assess robustness and generalisability of microbiome–caries associations using an integrated ONT-MET cross-platform design. Species consistently associated across platforms and analytical approaches were defined as robust candidate markers and replicated in two independent ONT-based cohorts. This approach identified reproducible microbiome-based markers across sequencing technologies, sampling methods, and DNA extraction methods, which might have future clinical utility in predicting caries risk.

## Material and methods

### Study groups

The Malmö Offspring Dental Study [8], with ONT and MET data, served as the derivation cohort (n_MODS-MET_ = 654 and n_MODS-ONT_ = 475). In MODS, 463 participants had complete paired MET, ONT, and dental data and were included in the cross-platform analyses (age 24–71 years). Replication analyses were conducted in STAGE (a sub-study of the Swedish Twin Register; age 23–52 years, *n* = 3,457 after selecting one twin per pair and single twins) and SMAC (age 16–23 years, *n* = 215), respectively. Both cohorts have been described previously [9,10]. A study flow chart is shown in Figure 1.

**Figure 1.**
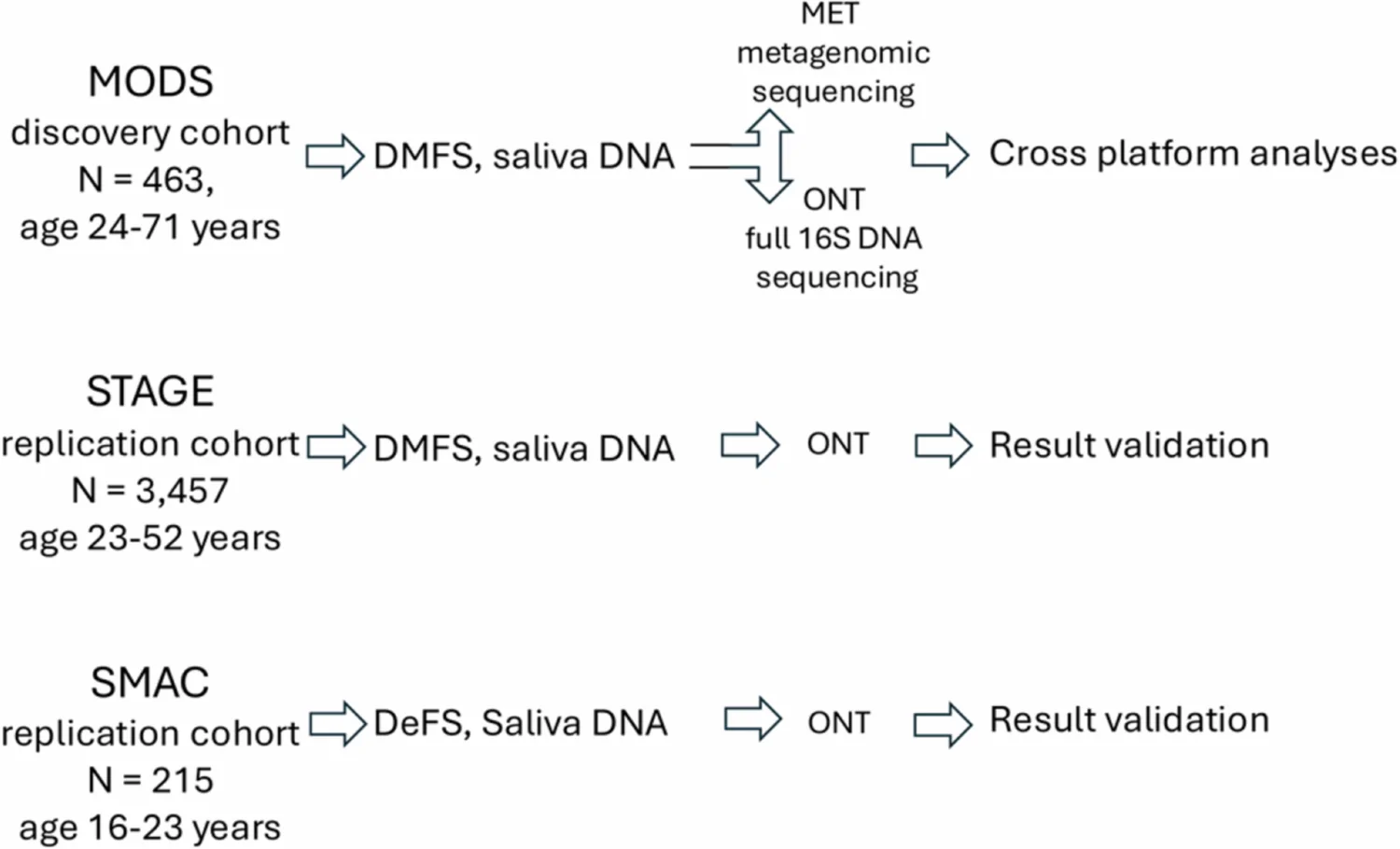
Overview of study flow and design.

This study was conducted in accordance with the Declaration of Helsinki including that all participants gave written informed consent. MODS was approved by the Ethical Review Board of Lund University (Dnr. 2013/761), STAGE and SMAC by the Swedish Ethics Review Authority (Dnr 2023-07530-01 and Dnr 09-134M, 2018/335-31, and 2023-07572-01).

### Dental examination

In MODS and SMAC, caries was assessed by study-specific clinical examinations (five calibrated dentists in MODS, inter-examiner agreement ≥90%; one dentist in SMAC), including saliva sampling, visual inspection, probing, and radiographs [8,10,11]. In STAGE, caries data within two years after saliva collection [11] were obtained from routine clinical dental examinations compiled and quality controlled in the Swedish quality register for caries and periodontal diseases (SkaPa) [12]. Tooth surfaces were classified as sound (D0); early enamel lesions, which may extend to the enamel-dentin junction (D1); lesions reaching the middle third of dentin (D2); cavitated lesions extending into the inner third of dentin (D3); or as filled (F) or missing (M). DMFS (D3, F, M) was used in MODS and STAGE, excluding early caries stages because STAGE included examinations performed by multiple dentists who were not formally calibrated. In SMAC, DeFS (D1–D3, F) was used to improve the outcome distribution; no teeth were missing due to caries, and all examinations were performed by the same dentist. Due to skewness, DeFS was Yeo–Johnson transformed using age- and sex-adjusted residuals [13]. Third molars were excluded.

### Saliva sampling and DNA extraction

In MODS and SMAC, chewing-stimulated saliva was collected, with participants refraining from toothbrushing and from eating or drinking (except water) for 1 hour prior to sampling, and stored at −80 °C until DNA extraction. For ONT, DNA was extracted using the GenElute™ Bacterial Genomic DNA kit (Sigma-Aldrich) with lysozyme, mutanolysin, and Proteinase K and RNase treatment and purification. For MET, DNA extraction was performed at Clinical Microbiomics A/S using the NucleoSpin 96 Soil kit [14]. In STAGE, participants collected unstimulated saliva at home using the Oragene OG-500 kit (DNA Genotek) and returned samples by mail. Here, saliva DNA was extracted using the Puregene blood kit (QIAgen, Hilden, Germany) or Chemagen (Revvity Chemagen Technology, Baesweiler, Germany). DNA quality and quantity were assessed using NanoDrop and Qubit instruments (Thermo Fisher Scientific) before sequencing.

### Sequencing

Full-length 16S rRNA gene sequencing using Oxford Nanopore Technologies (ONT) was performed as described previously [15]. Amplicons were PCR amplified, purified, barcoded, and sequenced on a GridION platform. Base-calling and demultiplexing used MinKNOW (SUP model), with reads filtered by quality (>12) and length (1,200–1,800 bp). Taxonomic annotation was performed using EMU against the eHOMD database [16,17].

Metagenomic sequencing was performed at Clinical Microbiomics A/S [14]. Libraries were prepared with NEBNext Ultra and sequenced on an Illumina NovaSeq 6000 (2 × 150 bp). Low-quality and host reads (GRCh38) were removed. Non-host reads were mapped to the Human Microbiome Reference (HMR05) gene catalogue [18], and species were defined by reads mapping to ≥3 signature genes, with taxonomy assigned using RefSeq-based criteria as defined by the company. For duplicate samples, the run with highest read depth was retained.

For both sequencing methods, positive (ZymoBIOMICS Microbial Community Standard DNA mock) and negative controls (ultrapure water) were included in each batch (Table S1). Species were retained if they were detected in at least two samples and had a cumulative read count of at least 25 across the entire dataset. Samples were retained if they contained at least 20,000 sequencing reads. For ONT, 509 of 551 sequenced samples met this criterion, and 519 named or eHOMD-defined species were retained. For MET, 705 of 706 sequenced samples met the same criterion, and 888 species were retained. A total of 463 samples met the sequencing-read criterion for both ONT and MET and were included in the cross-platform analyses.

### Met and ONT species harmonisation for comparative regression analyses

Unmatched metagenome-assembled genomes and genus-level taxa in MET and ONT were excluded, restricting analyses to named species which yielded 220 species identified by both platforms (Table S3).

### Statistical analyses

Participant characteristics were analysed using Stata 17.0 (StataCorp, College Station, TX). Descriptive analyses of microbial species, including binary detection (detected/not detected) and relative abundance following total sum scaling, and all subsequent microbiome analyses were performed in R (version 4.4.2) using the tidyverse, compositions, zCompositions and vegan packages. The analytical workflow comprised four sequential stages. First, ONT and MET sequencing data were compared to determine ecological and species-level concordance between platforms. Second, species associated with caries were identified independently in ONT and MET using abundance- and detection-based analyses. Third, species consistently associated with caries across platforms were combined into microbial risk scores. Finally, these scores were evaluated in two independent replication cohorts.

For descriptive abundance summaries and species-level cross-platform comparisons, raw species read counts were converted to relative abundances by total sum scaling, and mean abundance was calculated for each species as the arithmetic mean across all samples. For community-level concordance and abundance-based regression analyses, zero counts were replaced using multiplicative replacement before centred log-ratio (CLR) transformation. CLR-transformed abundances were used to calculate Aitchison distances [19] and as predictors in regression analyses.

Species prevalence was defined as the proportion of participants with a species read count greater than zero. To improve stability of regression estimates, species detected in fewer than 1% of participants were excluded from the regression analyses. The 1% threshold was selected after evaluating prevalence thresholds ranging from 0.1% to 5%, balancing retention of informative species against improved cross-platform agreement. This threshold retained 196 species for regression analyses (Table S2).

Concordance between ONT and MET was evaluated separately at the ecological and species levels. Ecological concordance was evaluated using three complementary measures: (i) Spearman correlation of paired Shannon diversity indices, (ii) Spearman correlation of pairwise inter-individual Aitchison distances derived from CLR-transformed abundances [20], and (iii) Procrustes analysis of principal coordinate ordinations derived from the Aitchison distance matrices. Species-level concordance was evaluated using abundance- and detection-based measures for named species identified by both sequencing platforms (Table S3). Abundance concordance was assessed using Spearman correlation of mean relative abundances. Detection agreement was evaluated as (i) species-wise proportion concordance, defined as the proportion of matched samples in which a species was detected by both platforms or absent from both platforms, and (ii) sample-wise Jaccard similarity coefficients calculated from binary presence/absence matrices.

Associations between microbial species and caries were evaluated using linear regression models adjusted for age, sex, and platform-specific sequencing depth. The outcome was continuous caries burden (DMFS in MODS and STAGE, and Yeo–Johnson transformed DeFS values in SMAC), with microbial predictors analysed as either binary detection variables (0 = absent; 1 = detected) or CLR-transformed abundances. False discovery rate correction was performed using the Benjamini–Hochberg procedure. Species were considered robust candidates if they fulfilled all of the following criteria: (i) concordant direction of association across MET and ONT sequencing platforms; (ii) FDR significance (q < 0.05) in both ONT and MET analyses using CLR-transformed abundances, together with supportive evidence from detection-based analyses (q < 0.1); and (iii) retention during nested cross-validation. The latter required a consistent effect direction across all cross-validation fits, *p* < 0.10 in ≥90% of fits, *p* < 0.05 in ≥85% of fits, mean *p* < 0.05, mean q ≤ 0.15, and non-zero prevalence ≥1%. Nested cross-validation was used to evaluate the stability of species selection rather than predictive performance. It comprised eight outer folds and five inner folds, with species-level regressions fitted within the inner training subsets of each outer training set.

Microbial risk scores were constructed from the robust candidate species. The detection-based score was calculated as the unweighted sum of detected species (Detection score = Σ Iᵢ), where Iᵢ = 1 if species *i* was detected and 0 otherwise. The CLR-based score was calculated analogously as the unweighted sum of the CLR-transformed abundances of the selected species (CLR score = Σ CLRᵢ). The CLR-based score was standardised as a z-score before inclusion in the regression analyses, whereas the detection-based score was analysed on its original scale, with a one-unit increase corresponding to one additional detected species. A coefficient-weighted CLR score, using regression coefficients estimated in the derivation cohort, was evaluated separately as a sensitivity analysis. A covariate-only base model containing age, sex, and sequencing depth was fitted for each cohort, with the detection-based or CLR-based score added in separate models. The incremental explanatory contribution of each score was quantified as the difference in R^2^ from the base model (ΔR^2^). Models containing both detection-based and CLR-based scores were also fitted, and likelihood ratio tests assessed the independent contribution of each score by comparing the combined model with models containing either score alone. Model stability was evaluated using non-parametric bootstrap resampling (500 replicates). In each iteration, participants were resampled with replacement and the base, CLR-score, and detection-score, models were re-fitted. Regression coefficients, R^2^, and ΔR^2^ were recorded and summarised as medians with empirical 95% confidence intervals.

Sensitivity analyses were performed to evaluate the robustness of the microbial risk scores. Less stringent selection criteria requiring only concordant direction of association across platforms yielded 14 positively associated and 12 negatively associated candidate species. Leave-one-species-out analyses were conducted by recalculating the scores after sequentially excluding one species at a time and comparing the resulting regression coefficients and explained variance (R^2^) with those obtained using the complete score. To assess whether negatively associated species contributed information beyond that provided by positively associated species, the risk scores were decomposed into positive-only, negative-only, and combined scores for the detection-based, unweighted CLR and weighted CLR models. Finally, overlap of candidate species identified across the three cohorts was examined, and the performance of a reduced score including only the five species identified in all cohorts was evaluated.

## Results

### Study group characteristics

The three study cohorts included both male and female participants in largely balanced proportions. Although the cohorts covered different age ranges, the mean number of teeth was similar, while caries scores were lower in the youngest cohorts. Detailed characteristics of the study populations are presented in Table 1.

**Table 1.** Study group characteristics.

	MODS *n* = 463	STAGE *n* = 3,457	SMAC *n* = 215
Sex, % females	49.7	61.6	54.6
Age, mean (SD)	53.6 (8.0)	37.3 (7.8)	17.8 (1.1)
BMI (kg/m^2^), mean (SD)	26.7 (4.3)	−	22.4 (3.2)
Number of teeth, mean (SD)	27.1 (1.7)	27.3 (1.4)	27.5 (1.5)
Bleeding on probing, mean (SD)	25.2 (17.4)	−	
Dental status			
DMFS, mean (SD)	36.0 (24.2)	17.9 (15.0)	−
DeFS, mean (SD)			4.3 (6.8)
FS, mean (SD)	30.8 (21.6)	14.1 (12.8)	−
MS, mean (SD)	4.4 (8.3)	3.6 (6.8)	0 (0)
D3, mean (SD)	0.59 (5.2)	0.12 (0.65)	−
D2, mean (SD)	0.75 (1.6)	0.93 (1.81)	−
D1, mean (SD)	0.68 (1.9)	1.8 (2.9)	−
D0, mean (SD)	102.5 (23.9)	107.4 (15.4)	−

### Cross-platform concordance of microbiome composition and ecological structure

We evaluated concordance in microbiota composition between ONT and MET in the MODS derivation cohort. As expected, sequencing depth differed between platforms, with higher detection rates for MET than ONT (Table S3). Despite these differences, multiple measures indicated moderate concordance in community-level characteristics between platforms. The distributions of *α*-diversity (Shannon index) broadly overlapped between platforms, with a narrower distribution for ONT (Figure 2A). Pairwise Shannon index values were moderately correlated (Spearman *ρ* = 0.50, *P* = 2.2 × 10⁻^16^), and inter-individual ecological distances based on CLR-transformed abundances were also moderately correlated (Spearman *ρ* = 0.48, *P* = 1 × 10⁻^4^; Figure 2B), indicating moderate concordance in inter-individual ecological relationships between platforms. This was further supported by Procrustes analysis of Aitchison-based ordinations (r = 0.60, *P* = 1 × 10⁻^4^), demonstrating alignment of community structure between platforms (Figure 2C). Detection-based profiles showed moderate agreement, with a median and mean Jaccard similarity of 0.54.

**Figure 2.**
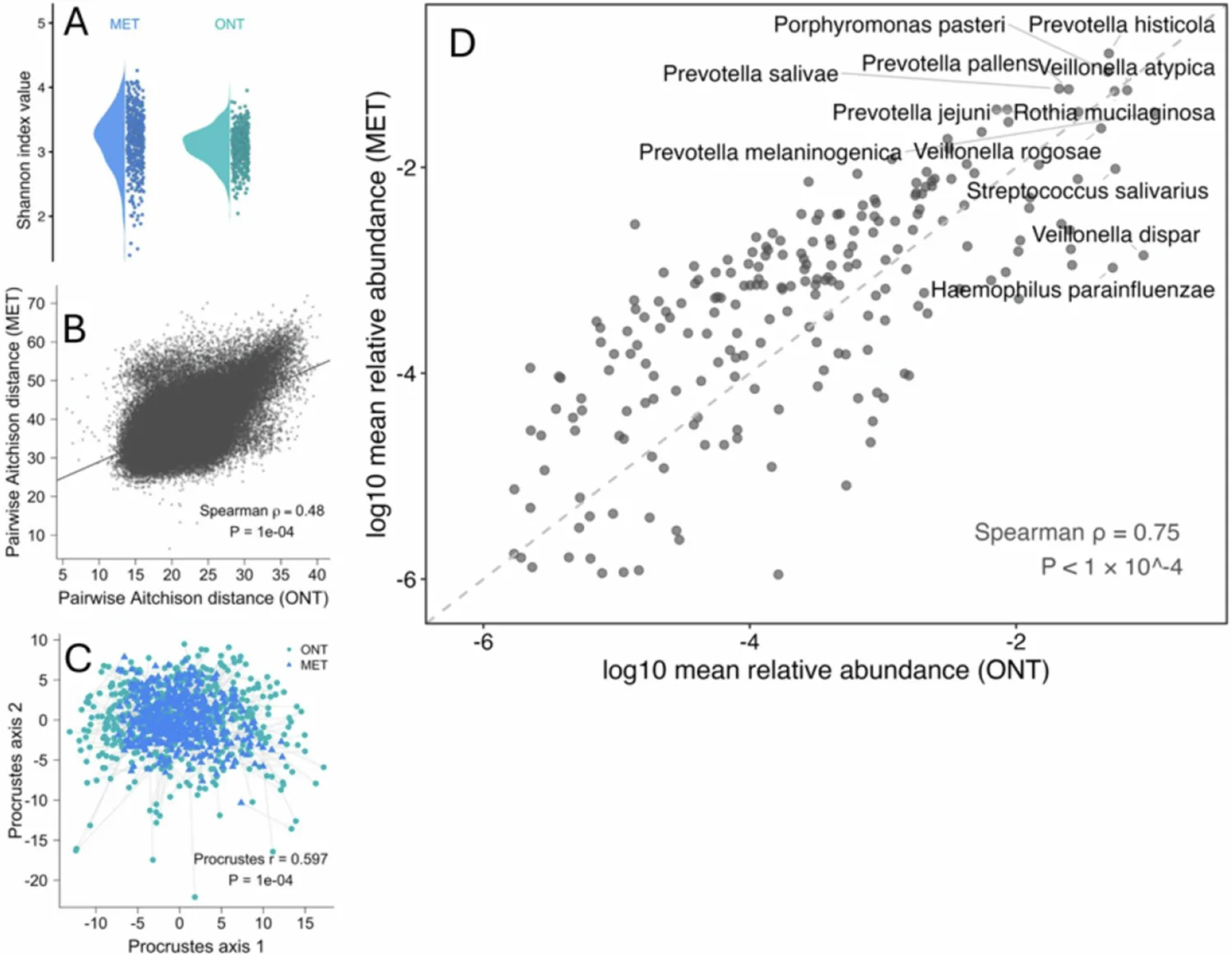
Cross-platform concordance of microbiome composition and ecological structure. (**A**) Comparison of *α*-diversity distribution by sequencing platforms measured by the Shannon index. Distributions for matched samples profiled by MET and ONT are shown, with slightly higher diversity observed in MET. (**B**) Correlation of pairwise ecological dissimilarities between samples based on Aitchison distances derived from CLR-transformed species abundances. Each point represents a pairwise comparison between samples. A moderate but significant correlation (Spearman *ρ* = 0.49, *P* = 1 × 10⁻^4^) indicates that inter-individual ecological relationships are broadly preserved across platforms. (**C**) Procrustes analysis of principal coordinate ordinations based on Aitchison distances derived independently from MET and ONT data. The significant alignment between configurations (r = 0.609, *P* = 1 × 10⁻^4^) demonstrates concordance in global community structure despite platform-specific differences. (**D**) Correlation of mean relative abundances of shared species between MET and ONT. Mean relative abundances are log-transformed, and each point represents a species, with the top 12 species indicated. A strong correlation (Spearman *ρ* = 0.75, *P* < 1 × 10⁻^4^) indicates high agreement in average abundance patterns across species. In contrast, agreement for individual species across samples is more variable, with detection-based measures (presence/absence) generally showing higher concordance than CLR-transformed abundances (Table S3).

At the species level, mean relative abundances were strongly correlated (Spearman *ρ* = 0.75, *P* < 1 × 10⁻^4^; Figure 2D). However, agreement for individual species across samples was more variable, with detection-based measures generally showing higher concordance than CLR-transformed abundances (Table S4), indicating that platform effects were more apparent in quantitative species-level abundance estimates. Together, these findings indicate that while ONT and MET differ in sequencing depth and species-specific signals, they capture broadly consistent ecological structure and overall abundance patterns of the oral microbiome.

### Identification of potential caries-associated taxa

To identify robust caries-associated species, cross-platform consistency was evaluated by comparing species-specific associations with DMFS across both sequencing platforms. Statistical significance (q-value < 0.05) was required in both platforms. Robustness was further assessed using nested cross-validation and complementary detection-based (presence/absence) modelling. All models were adjusted for age, sex, and sequencing depth.

Among the 196 retained taxa, 14 species were significantly associated with DMFS based on CLR-transformed abundance in both platforms, using less stringent selection criteria (both q < 0.05). When applying the stricter cross-validation criteria, four species were not supported, resulting in a final set of 10 candidate species (Figure 3). When evaluating additional caries phenotypes, no species was consistently associated with early enamel lesions (D1), and only one species (*Lactobacillus gasseri*) was associated with D2 lesions. In contrast, a larger number of species were associated with advanced lesions (D3), indicating that salivary microbiome shifts become more pronounced with lesion progression. Associations for untreated caries and filled surfaces largely overlapped with those identified for DMFS/DeFS, whereas D0 (caries-free surfaces) showed a distinct microbial profile of which several are non-saccharolytic species, such as *Catonella morbi, Fusobacterium periodonticum, Porphyromonas pasteri,* and *Treponema vincentii* (Figure 3).

**Figure 3.**
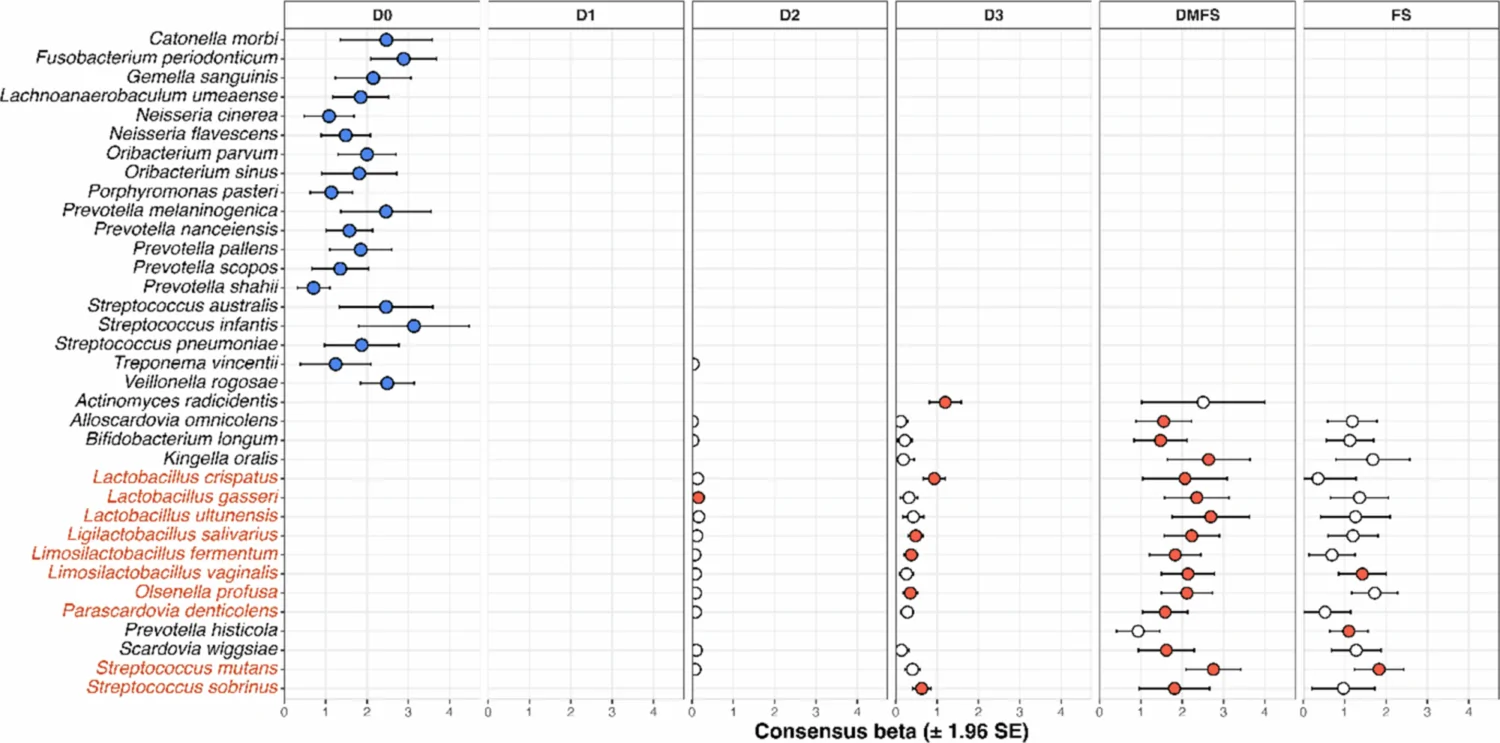
Forest plot of cross-platform consensus associations between microbial species and caries-related phenotypes. Species positively associated with different measures of caries status are shown based on analyses of the 463 MODS saliva samples. Filled red symbols indicate species with concordant significant associations in both MET and ONT analyses (FDR q < 0.05), whereas unfilled symbols indicate species significant in only one platform. Taxa retained after application of the additional cross-validation filter and included in the final 10-species risk score are indicated by red species names on the y-axis. Points represent regression coefficients (± 1.96 SE). Associations for D0 (caries-free surfaces) are shown in blue. D1, D2, and D3 represent increasing severity of caries, from initial enamel changes to cavitated lesions. FS denotes the number of filled surfaces, and DMFS the cumulative number of decayed, missing, and filled surfaces.

The prevalence and mean relative abundance of the 10 candidate species varied across cohorts and sequencing platforms (Figure 4). Several species showed lower prevalence and relative abundance in STAGE and SMAC than in MODS.

**Figure 4.**
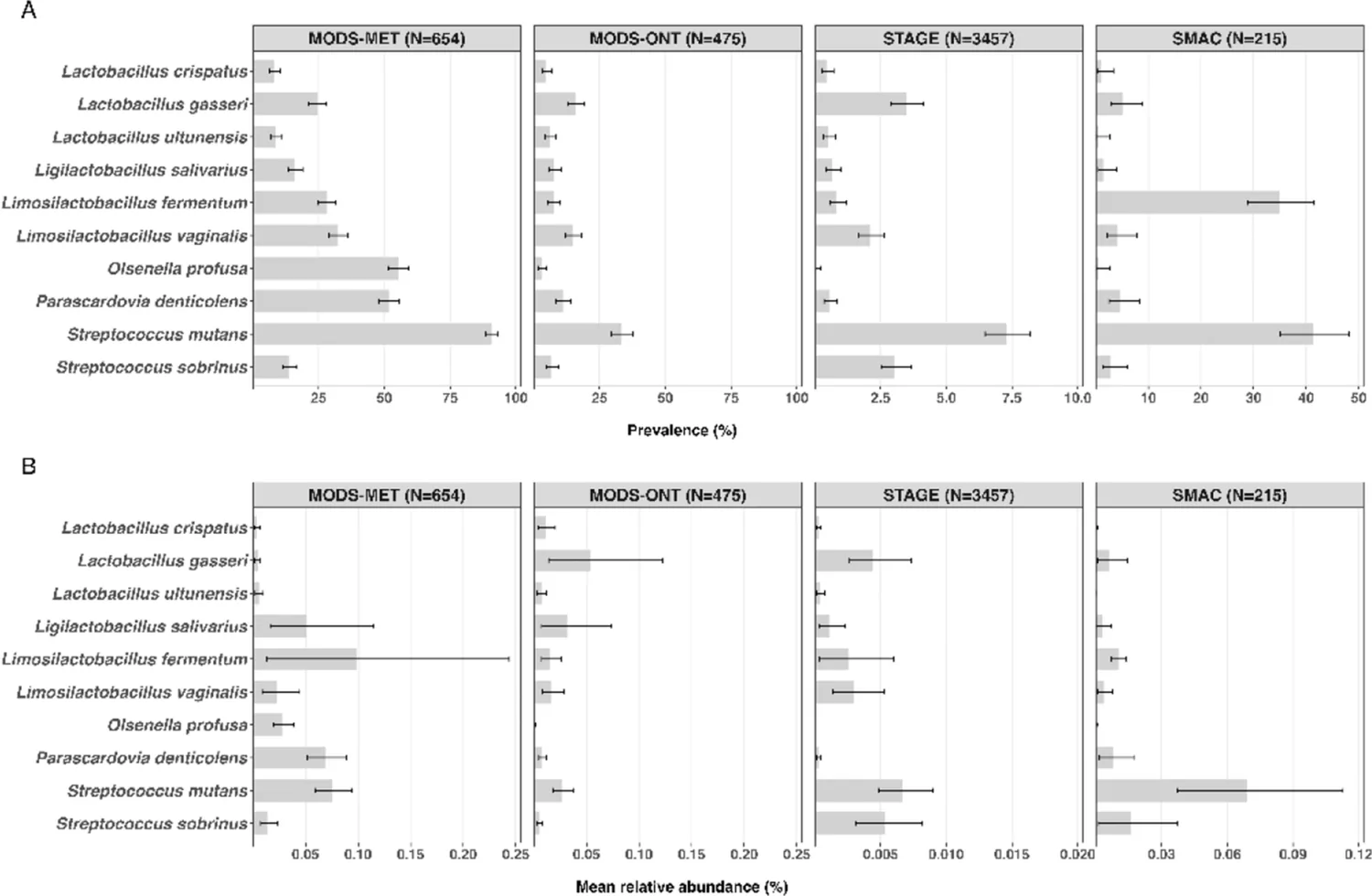
Prevalence and mean relative abundance of the ten species comprising the microbial risk score across cohorts and sequencing platforms. **(A)** Species prevalence, defined as the proportion of samples in which the species was detected (>0 reads), with 95% Wilson confidence intervals. **(B)** Mean relative abundance, calculated by total-sum scaling of raw read counts and averaged across all samples, including samples in which the species was not detected, with bootstrap 95% confidence intervals based on 2,000 resamples. Results are shown separately for MODS-MET, MODS-ONT, STAGE, and SMAC and employ all cohort members. Bars represent point estimates and horizontal whiskers the corresponding 95% confidence intervals. Cohort-specific linear x-axis ranges were used for STAGE and SMAC to improve visualisation of their lower prevalence and relative abundance estimates; note that x-axis scales therefore differ between cohorts.

### Construction and performance of microbial risk scores

Microbial risk scores incorporating the 10 candidate species were constructed using two approaches: (i) abundance-based scores derived from CLR-transformed values and (ii) detection-based scores reflecting the number of detected species. Both unweighted and effect size-weighted versions were evaluated. ONT-based scores were generally lower than MET-based scores, with the lowest values observed in STAGE (Figure 5A), paralleling increasing proportions of individuals with zero scores (MODS-MET 3.5%, MODS-ONT 57.7%, SMAC 37.0%, STAGE 87.5%). Despite these differences, CLR- and detection-based scores showed similar explanatory power in models adjusted for age, sex, and sequencing depth (R^2^ 0.404-0.456). In contrast, effect sizes were expressed differently for the two scores; CLR-based scores indicated an increase of 6–7 DMFS surfaces per standard deviation, whereas detection-based scores indicated an increase of approximately 4 surfaces per additional detected species (Table S5). In sensitivity analyses, associations were consistent across sex but with some differences by age (Table S5). Replication in STAGE and SMAC confirmed similar within-cohort performance, although overall R^2^ values were lower than in MODS (STAGE mean R^2^ = 0.27, SMAC mean R^2^ = 0.10). All associations remained statistically significant (Table S5). Effect size weighting did not materially improve model performance (Table S6). While small increases in explained variance were observed in the derivation cohort (MODS), no improvement was seen in STAGE and performance decreased in SMAC. For CLR-based scores, weighting likewise provided no consistent benefit.

**Figure 5.**
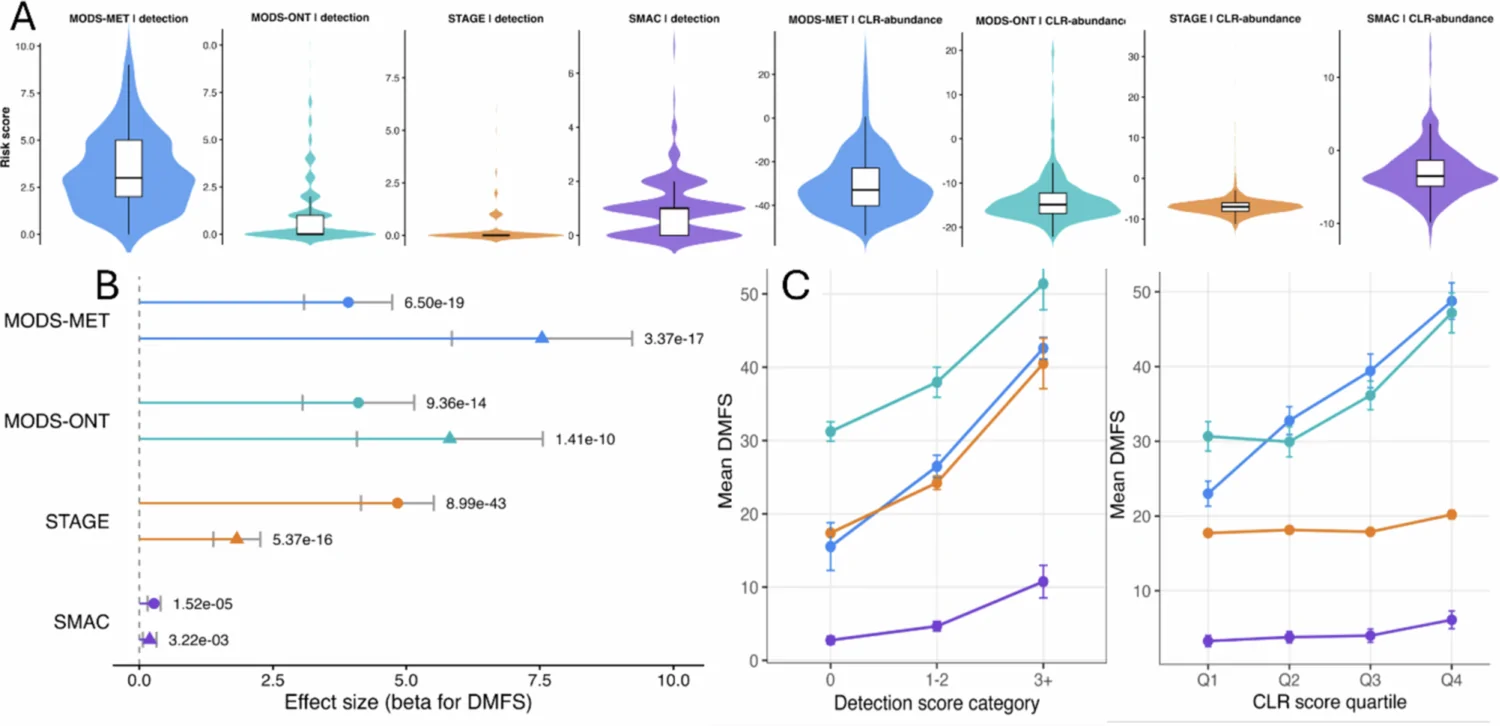
Overview of microbiota-based risk score and caries burden. For the different settings (**A**) shows the distribution of microbiota-based risk scores; (**B**) lollipop plot illustrating the effect sizes (beta values) from linear regressions including sex, age and number of sequencing reads; and (**C**) DMFS/DeFS values per risk score value.

Including both score types yielded only modest increases in explained variance compared with detection-only models (ΔR^2^ ≈ 0.004–0.005), with moderate collinearity (VIF ≈ 2.1–3.6). Likelihood ratio tests further showed that detection-based scores contributed more independently to model fit than CLR-based scores (MET: *P* = 6.4 × 10⁻^5^ vs 0.009; ONT: *P* = 9.03 × 10⁻^5^ vs 0.050) (Table S7).

### Sensitivity and robustness analyses

Leave-one-out analyses showed that associations between the microbial risk score and caries burden were robust to exclusion of individual species, with only minor changes in effect estimates and explained variance across cohorts (Table S8).

To assess whether negatively associated species contributed information beyond positively associated species, risk scores were decomposed into positive-only, negative-only, and combined scores for detection-based, unweighted CLR, and weighted CLR models (Table S9). Across all cohorts, the positive detection-based score explained more variance than the negative-species score alone, and combining positively and negatively associated species did not materially improve model performance. Similarly, for both unweighted and weighted CLR models, positive-only and combined scores showed virtually identical explanatory power. In the weighted CLR models, the positive and negative components were highly correlated (r = 0.98). A species-level correlation heatmap supported this redundancy, with positively and negatively associated species forming separate clusters and correlations between the two groups being largely inverse (Figure S1). Together, these findings suggest that positively and negatively associated species largely reflect a shared ecological gradient rather than independent predictive components.

Per-cohort identification of candidate species showed that seven of the ten species identified in MODS were also identified in STAGE, and five were consistently identified across all cohorts (Table S10). A reduced score based on these five species showed comparable explanatory power to the full 10-species score, with only minor differences in R^2^, but with a higher proportion of individuals assigned a score of zero. Together, these findings indicate that the microbial signal is partially redundant across species, but that reducing the number of species increases sensitivity to detection differences across cohorts and platforms.

## Discussion

In this study, we evaluated consistency in oral microbiome profiling across sequencing platforms and identified a reproducible microbial signature associated with dental caries across multiple adult cohorts. Although overall community structure showed moderate concordance between platforms and mean relative abundances of species were strongly correlated, agreement for individual species across samples was more variable. These findings highlight the importance of distinguishing between community-level patterns and species-level estimates when comparing sequencing results. Within this framework, we identified caries-associated species which were consistent across platforms and cohorts and derived a microbial risk score that replicated in independent populations, supporting a multi-species detection-based score as a simple, robust, and interpretable summary of microbial risk for caries.

Several caries-associated species were consistently identified across sequencing platforms and cohorts despite differences in sequencing depth, taxonomic resolution, and bioinformatic processing. Using these reproducible species, we developed microbial risk scores based on species detection and CLR-transformed abundance. Both score types were consistently associated with caries in the derivation and the two independent replication cohorts, although detection-based scores performed slightly better. Combining species detection and CLR-transformed abundance in the models yielded only modest improvements, indicating substantial overlap in the information captured by the two approaches. Likelihood ratio tests further demonstrated that detection-based scores contributed more independently to model fit. Species-level associations with caries were strongest for advanced lesions, whereas relatively few species were positively associated with early enamel changes, suggesting that microbiome shifts become more distinct with disease progression [21]. This is consistent with the ecological plaque hypothesis, whereby prolonged acidification favours increasingly specialised acidogenic and aciduric communities, resulting in stronger and more reproducible microbial associations in advanced caries.

Dental caries is widely recognised as a polymicrobial disease characterised by ecological shifts rather than single pathogens. Consistent with this, leave-one-out analyses showed that associations between the microbial risk score and caries burden were robust to exclusion of individual species, with only minor changes in effect estimates and explained variance across cohorts. Classical cariogenic species such as *Streptococcus mutans* and *Streptococcus sobrinus*, together with acidogenic and aciduric species including lactobacilli and bifidobacteria, have repeatedly been linked to disease [22]. Several members of the *Lactobacillaceae* family were identified despite often being low-abundance taxa, which may contribute to their underrepresentation in studies applying stringent prevalence thresholds [23]. Previous approaches to quantifying microbial contributions to caries risk have relied on salivary counts of mutans streptococci and lactobacilli [24]. However, in contrast to periodontitis, where Socransky’s microbial complexes are well-established disease signatures [25], no broadly accepted or reproducible microbial risk score exists for dental caries. Our findings extend this literature by demonstrating that a simple detection-based multi-species score captures a robust and reproducible microbial signal across sequencing platforms and independent cohorts.

In contrast, association of several anaerobic, proteolytic, and periodontitis-associated species among caries-free individuals (D0) is consistent with a concept that microbial profiles associated with low caries activity may partly overlap with a periodontitis-type ecology [26]. However, this should be interpreted as an ecological pattern rather than evidence of increased periodontal disease risk unless supported by further clinical associations.

The risk score was associated with caries across cohorts despite differences in age, saliva collection procedures, DNA extraction methods, and caries phenotyping. The younger SMAC cohort and the STAGE cohort, which used unstimulated saliva collected outside the clinical setting, differed substantially from the derivation cohort and may partly explain the attenuation of associations observed in the replication analyses. Methodological differences may also influence microbial detection and taxonomic resolution, particularly for Gram-positive taxa, whose recovery can vary depending on DNA extraction and cell lysis procedures. In addition, the use of different caries phenotypes (DMFS versus DeFS) may have affected effect size estimates, as inclusion of early enamel lesions may capture earlier and potentially more transient stages of disease that are less strongly associated with stable microbial shifts. Nevertheless, the persistence of significant associations across cohorts suggests that the identified microbial signature is robust to both technical and biological variation. Weighting scores by effect size did not improve model performance overall, with small gains limited to the derivation cohort and no improvement or reduced performance in replication cohorts. Similarly, alternative score constructions incorporating positively and negatively associated species yielded highly similar model performance, indicating overlapping information. Together, these findings indicate that the microbial signal is distributed across species and robust to model specification, with limited benefit from increased model complexity.

Strengths of this study include matched samples across sequencing platforms and independent cohorts for replication. The combination of compositional data analysis, cross-platform validation, and cross-validated modelling provides a robust framework for identifying reproducible microbial associations. Limitations include the use of relatively homogeneous Swedish cohorts, which may limit generalisability. The cross-sectional design precludes causal inference, and observed associations may reflect both causes and consequences of disease. Although associations were consistent across cohorts, formal interaction analyses were limited, and the comparably small derivation cohort may limit model stability and the precision of R^2^ estimates. However, the results should be interpreted in the context of replication and generalisability rather than as direct comparisons of predictive accuracy. In addition, harmonised information on several behavioural and environmental factors, including diet, oral hygiene, fluoride exposure, smoking, medication use, and socioeconomic status, were not available across all cohorts. Residual confounding by these factors cannot therefore be excluded, although the consistency of findings across independent cohorts suggests that the observed associations are unlikely to be entirely attributable to cohort-specific confounding structures. Hence, validation in more diverse populations is warranted. Furthermore, the study was designed to identify reproducible microbial associations with continuous measures of caries burden rather than to evaluate diagnostic performance, and the proposed score should therefore not be interpreted as a clinically validated diagnostic or risk prediction tool.

## Conclusions

This study shows that key features of the oral microbiome are reproducible associated across sequencing platforms, with consistent abundance patterns among dominant shared species. We identified a robust microbial signature of caries and derived a risk score capturing the combined presence of caries-associated species. These findings support the development of platform-independent microbial markers while highlighting the importance of accounting for methodological variability in species-level analyses. Although deeper sequencing increased detection sensitivity and reduced the proportion of individuals with zero scores, the overall microbial signal remained reproducible across platforms. Future studies may evaluate the discriminatory diagnostic power and clinical usefulness of the score in longitudinal settings.

## Supplementary Material

Supplementary Material.xlsx Supplementary Material.xlsx

## Data Availability

Metadata are provided in the Supplementary Tables. Individual-level data cannot be made publicly available due to restrictions under the General Data Protection Regulation (GDPR) and Swedish data protection legislation, as the data contain sensitive personal information. Data may be made available to qualified researchers upon reasonable request addressed to Dr. Daniel Jönsson and Dr. Anders Esberg, subject to approval by the Swedish Ethical Review Authority.
